# Effect of Substrate Temperature on the Structural, Morphological, and Infrared Optical Properties of KBr Thin Films

**DOI:** 10.3390/ma18153644

**Published:** 2025-08-03

**Authors:** Teng Xu, Qingyuan Cai, Weibo Duan, Kaixuan Wang, Bojie Jia, Haihan Luo, Dingquan Liu

**Affiliations:** 1 Shanghai Key Laboratory of Optical Coatings and Spectral Modulation, Shanghai Institute of Technical Physics, Chinese Academy of Sciences, Shanghai 200083, China; xuteng2023@shanghaitech.edu.cn (T.X.); qycai@mail.sitp.ac.cn (Q.C.); duanweibo@mail.sitp.ac.cn (W.D.); wangkx@shanghaitech.edu.cn (K.W.); jiabojie22@mails.ucas.ac.cn (B.J.); 2 School of Physical Science and Technology, ShanghaiTech University, Shanghai 200031, China; 3 School of Optoelectronics, University of Chinese Academy of Sciences, Beijing 100049, China

**Keywords:** potassium bromide, thin film, substrate temperature, structural properties, infrared optical properties, resistive evaporation

## Abstract

Potassium bromide (KBr) thin films were deposited by resistive thermal evaporation at substrate temperatures ranging from 50 °C to 250 °C to systematically elucidate the temperature-dependent evolution of their physical properties. Structural, morphological, and optical characteristics were examined by X-ray diffraction (XRD), scanning electron microscopy (SEM), atomic force microscopy (AFM), and Fourier transform infrared spectroscopy (FTIR). The results reveal a complex, non-monotonic response to temperature rather than a simple linear trend. As the substrate temperature increases, growth evolves from a mixed polycrystalline texture to a pronounced (200) preferred orientation. Morphological analysis shows that the film surface is smoothest at 150 °C, while the microstructure becomes densest at 200 °C. These structural variations directly modulate the optical constants: the refractive index attains its highest values in the 150–200 °C window, approaching that of bulk KBr. Cryogenic temperature (6 K) FTIR measurements further demonstrate that suppression of multi-phonon absorption markedly enhances the infrared transmittance of the films. Taken together, the data indicate that 150–200 °C constitutes an optimal process window for fabricating KBr films that combine superior crystallinity, low defect density, and high packing density. This study elucidates the temperature-driven structure–property coupling and offers valuable guidance for optimizing high-performance infrared and cryogenic optical components.

## 1. Introduction

Infrared (IR) optical coatings are indispensable in windows, antireflection (AR) stacks, bandpass filters, and beam splitters. They ultimately dictate the performance of individual components and, by extension, entire optical systems [1,2]. In this context, potassium bromide (KBr), a classical alkali halide material, combines an exceptionally wide transmission window (approximately 0.25–25 µm) with a low refractive index (n ≈ 1.5). These attributes make KBr uniquely attractive for multilayer AR coatings, broadband IR windows, and cryogenic detector assemblies. When compared to common IR materials like ZnS, ZnSe, and Ge, the lower refractive index of KBr inherently reduces surface reflectance and simplifies the design of impedance-matching layers. Its lower density and cost further enhance its appeal for applications in large-aperture, weight-constrained optics. Therefore, a systematic investigation of KBr thin film processing to establish clear structure–property relationships is a critical step toward realizing the next generation of high-performance IR components.

A variety of techniques have been employed for the fabrication of KBr thin films [3,4,5]. Among these, thermal evaporation—and specifically resistive evaporation—is widely utilized due to its process maturity, capacity for precise stoichiometric control, and the inherently low optical absorption of the resulting films [6,7]. Existing research on KBr, in both bulk and thin film forms, has yielded significant insights. For instance, in the FIR region, Johnson and Bell rigorously determined the optical constants (n and k) of bulk KBr using asymmetric Fourier transform spectroscopy [8], with subsequent work by Hadni et al. extending these characterizations to other spectral intervals [9]. For thin films, the work of Rai et al. is notable; they utilized XRD, SEM, and AFM to establish correlations between film thickness and key properties such as crystallinity, crystallite size, surface morphology, and photoemission yield. Their findings consistently demonstrated that structural quality and photocurrent improve with increasing film thickness [10,11,12]. Other studies have focused on nascent film growth, with Alidjanov et al. probing the early stages on Si(111) to estimate the critical island nucleus size [13]. Furthermore, the stability and performance of KBr films under external stimuli have been a key area of investigation, particularly concerning their prospective use in photocathodes and far-IR filter assemblies [14,15,16,17]. Critically, Tremsin and Siegmund reported that heating the substrate to approximately 90 °C markedly enhances the UV-radiation tolerance of KBr coatings [18]. This particular finding underscores the pivotal role of deposition temperature in tuning film properties.

Despite this progress, a quantitative understanding of how substrate temperature governs the microstructure and, in turn, the optical properties of KBr films remains incomplete. While a few studies note that moderate heating can improve film stability, comprehensive datasets linking deposition temperature to broadband IR optical constants are scarce. Furthermore, optical characterization under cryogenic conditions (e.g., 6 K), critical for certain applications, is virtually absent. This knowledge gap limits a full mechanistic understanding of KBr film growth and hinders the precision tuning of its optical performance, thereby constraining the material’s potential in advanced IR systems.

To address this gap, we systematically investigated KBr films deposited by resistance evaporation at substrate temperatures ranging from 50 °C to 250 °C. We employed a suite of characterization techniques (XRD, SEM, AFM) to quantify temperature-driven changes in the film’s crystal structure, micro-stress, and surface morphology. Correspondingly, Fourier transform IR spectroscopy (FTIR) was used to extract the optical constants, revealing the underlying structure–property coupling. This work aims to establish a quantitative framework linking deposition temperature with microstructural and optical properties to enable predictive design of high-performance KBr-based IR coatings.

## 2. Experimental Details

### 2.1. Sample Preparation

KBr thin films, with a nominal thickness of 1.5 µm, were prepared by resistive evaporation onto various substrates, including single-crystal silicon (100) and diamond wafers. The evaporation source consisted of high-purity KBr powder (99.99%) contained within a molybdenum boat. Prior to deposition, all substrates underwent a sequential ultrasonic cleaning procedure: 10 min in a mixed solution of ethanol and diethyl ether (1:1 *v*/*v*), followed by 10 min in deionized water. The cleaned substrates were then dried using a stream of dry nitrogen before being loaded into the vacuum chamber. The temperature of the substrate holder was monitored and controlled by a K-type thermocouple attached in close proximity to the substrates. During evaporation, the chamber pressure was maintained at 2–4 × 10^−3^ Pa. For all depositions, the deposition rate was controlled at 40 Å/s, and the substrate holder was rotated at 60 rad/min to ensure film uniformity. An INFICON IC/5 deposition controller (INFICON, Syracuse, NY, USA), integrated with a quartz crystal microbalance, was used for in situ monitoring and control of the deposition rate and film thickness. The substrates were mounted on a stainless-steel holder and heated by quartz lamps to a series of designated temperatures: 50, 100, 150, 200, and 250 °C. To ensure thermal equilibrium, the substrates were held at the setpoint temperature for two hours before deposition commenced. Following deposition, the samples were immediately stored in a sealed container with desiccant and characterized promptly to minimize any effects from ambient exposure.

### 2.2. Film Characterization

The crystal structure of the KBr films deposited on Si (100) substrates was investigated using an X-ray diffractometer (D8 ADVANCE, Bruker, Karlsruhe, Germany). The system was operated in the Bragg–Brentano (θ–2θ) parafocusing geometry with a Cu Kα radiation source (λ = 1.5406 Å). Diffraction patterns were recorded at room temperature (25 °C) in continuous-scan mode across a 2θ range of 20°–65°. Surface and cross-sectional morphologies of the films were examined by field emission scanning electron microscopy (FE-SEM; SU9000, Hitachi, Tokyo, Japan) at accelerating voltages between 5 and 10 kV. The surface topography and root-mean-square (RMS) roughness were quantified over 5 µm × 5 µm areas using an atomic force microscope (AFM; VERO, Oxford Instruments, Abingdon, UK). Infrared transmittance spectra were acquired using a Fourier transform infrared (FTIR) spectrometer (Spectrum GX, PerkinElmer, Waltham, MA, USA). For measurements in the 1.2–6 µm range, films on silicon substrates were used. For the 5–28 µm range and cryogenic measurements, films deposited on diamond substrates were utilized to leverage diamond’s broad infrared transparency. For measurements at room temperature, spectra were recorded in ambient air. For cryogenic measurements, the sample was mounted on a copper cold finger inside a liquid helium optical cryostat under high vacuum and stabilized at 6 K before acquiring the spectra in situ. Following data acquisition, the refractive index (n) and physical thickness (d) of the films were extracted by fitting the experimental spectra with the Film Wizard™ software (version 6.10.1) package. Within the fitting model, the wavelength-dependent refractive index, n(λ), was described by the Cauchy dispersion formula,

(1) $$n\left(\lambda \right)=A+\frac{B}{\lambda^{2}}+\frac{C}{\lambda^{4}}$$

where λ is the vacuum wavelength, and A, B, and C are the Cauchy coefficients determined from the fitting process.

## 3. Results and Discussions

### 3.1. Structural Properties

**Figure 1:**
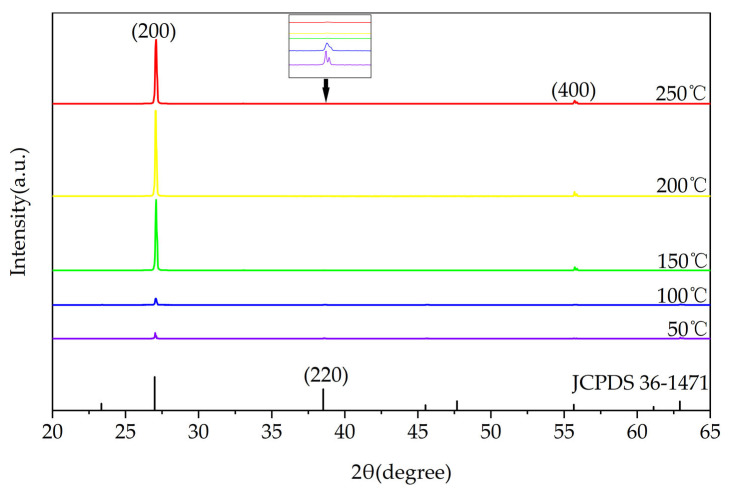
XRD patterns of KBr thin films deposited at different substrate temperatures.

The X-ray diffraction (XRD) patterns of the deposited KBr films are presented in Figure 1. All films, regardless of deposition temperature (50–250 °C), exhibit the face-centered cubic (fcc) structure characteristic of bulk KBr (JCPDS No. 36-1471), with the (200) diffraction peak at 2θ ≈ 27° being dominant. A clear temperature-dependent evolution of the film texture is observed. At lower temperatures, a secondary (220) peak at 2θ ≈ 38° is also evident. As the substrate temperature increases, this (220) peak is progressively suppressed, while the intensity of the (200) peak grows significantly. This trend signifies a systematic texture transition toward a preferential <100> orientation. The transition culminates at temperatures of 150 °C and above. In this regime, the films exhibit only the strong (200) reflection and its higher-order (400) counterpart (at 2θ ≈ 55°), providing unambiguous evidence of a highly (200)-oriented structure. This texturing behavior is consistent with thermodynamic principles for NaCl-type crystals. The enhanced adatom mobility at elevated temperatures allows the electrically neutral, lowest-energy {100} planes to align parallel to the substrate surface during growth.

The preferred orientation of film growth can be determined quantitatively by calculating the texture coefficient (TC*_(hkl)_*) along diffraction planes. The (TC*_(hkl)_*) of each (*hkl*) plane is evaluated from the XRD data according to the following formula [19].

(2) $${TC}_{\left(hkl\right)}=\frac{I_{\left(hkl\right)}/I_{(hkl)0}}{N^{-1}\sum_{N}I_{\left(hkl\right)}/I_{\left(hkl\right)0}}$$

where I*_(hkl)_* is the measured XRD peak intensity; I*_(hkl)_*_0_ is the intensity of randomly oriented KBr powder taken from a standard reference data; and N is the total number of diffractions under consideration.

The interplanar spacing, d, was calculated using Bragg’s Equation [20], as follows:

(3) 2dsinθ=nλ

where θ is the glancing angle and λ is the wavelength of the incident X-rays (=1.54 Å). In the cubic lattice, the lattice constant, a, is as follows:

(4) $$a=d\sqrt{h^{2}+k^{2}+l^{2}}$$

where h, k, and l are the Miller indices. The stress in the films was calculated from the equation [21], as follows:

(5) $$s=\frac{\left(a_{0}-a\right)Y}{2a_{0}\sigma }$$

where a_0_ and a are the lattice parameters in bulk and thin film form of KBr. Y is the Young’s modulus (=26.8 Gpa) and σ is the Poisson’s ratio (=0.203) for KBr, respectively. The strain in thermally deposited films was calculated from the following equation:

(6) $$\varepsilon =\frac{\beta }{4\tan\theta }$$

where β is the full width at half maximum (FWHM). The value of β was corrected for the instrumental broadening, which was determined from the FWHM of the primary diffraction peak of a standard, strain-free silicon powder sample. The crystallite size was calculated using the following equation [22]:

(7) $$D=\frac{0.9\lambda }{\beta \cos\theta }$$

The dislocation density is given by the following equation:

(8) $$\delta =\frac{1}{D^{2}}$$

**Figure 2:**
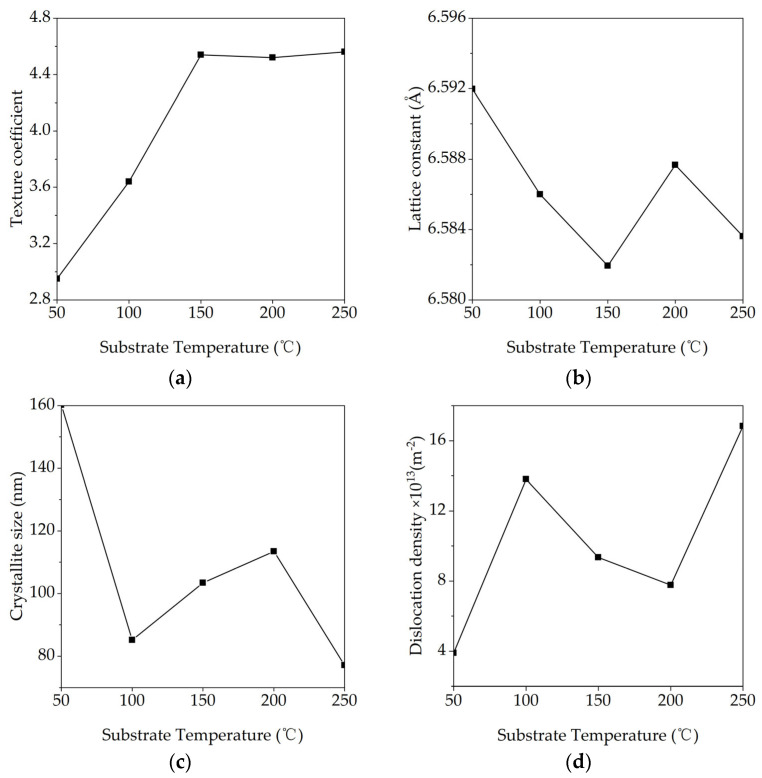

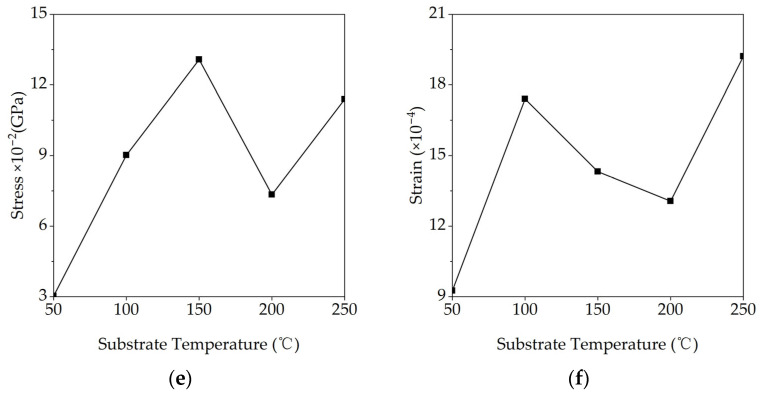
Variation in (**a**) texture coefficient, (**b**) lattice constant, (**c**) crystallite size strain, (**d**) dislocation density, (**e**) stress, and (**f**) strain of KBr films with substrate temperature.

The aforementioned parameters were found to vary with the substrate temperature. Therefore, XRD analysis was performed on the films deposited at different substrate temperatures, and the resulting patterns are plotted in Figure 2.

Figure 2a reveals a pronounced temperature dependence of the (200) texture coefficient, TC(200). A pronounced increase is observed initially, with the coefficient rising from 2.95 at 50 °C to a peak of 4.54 at 150 °C. Beyond this temperature, the TC(200) value enters a saturation regime, fluctuating by less than ±0.05. This trend indicates that enhanced surface diffusion at elevated temperatures promotes the rapid expansion of low-energy {100} facets, thereby converting the film from a multi-oriented structure into a saturated <100> fiber texture. The saturation above 150 °C suggests that most crystallites are already realigned, with further heating primarily promoting defect annihilation and stress relaxation rather than increasing the degree of texturing.

Crystallite size, derived from the Scherrer analysis of the (200) peak (Figure 2c), shows a non-monotonic trend. Large domains (160 nm) formed at 50 °C shrink abruptly to 85 nm at 100 °C owing to a nucleation burst triggered by the modest rise in adatom mobility. Subsequent heating to 150 °C and 200 °C activates boundary migration and coalescence, enlarging the domains to 103 nm and 113 nm, respectively. However, at 250 °C, the size falls again to 77 nm. This reversal is attributed to the onset of mild KBr re-evaporation, coupled with thermally induced stress that can fragment the lattice. Such oscillatory behavior, where initial growth is eventually counteracted by re-evaporation and stress dynamics, is consistent with reports on other evaporated polycrystalline films like ZnO, CdS, and SnS thin films [23,24,25].

The evolution of other microstructural parameters aligns with these findings. Dislocation density varies inversely with crystallite size, following a “rise–fall–rise” sequence. The initial temperature increase refines crystallites and raises boundary area, fostering dislocation generation; the 150–200 °C window encourages defect recombination and lowers the density; renewed re-evaporation and thermal stress at 250 °C regenerate defects. The lattice parameter, residual stress and micro-strain evolve cooperatively: the lattice constant contracts from low to mid temperatures, partially relaxes near 200 °C, and contracts again at 250 °C, while residual stress and micro-strain exhibit a complementary “increase–decrease–increase” profile [26]. Collectively, these findings identify the 150–200 °C range as the optimal processing window for producing KBr films with superior structural quality, characterized by low dislocation density, minimal residual stress/strain, and high crystallinity. This conclusion is directly corroborated by subsequent SEM observations. Furthermore, these distinct microstructural differences are expected to govern the infrared transmittance and optical constants discussed in Section 3.3.

### 3.2. Morphological Properties

This section investigates the influence of substrate temperature on the microstructure of KBr thin films to elucidate the critical role of temperature during the film growth process.

**Figure 3:**
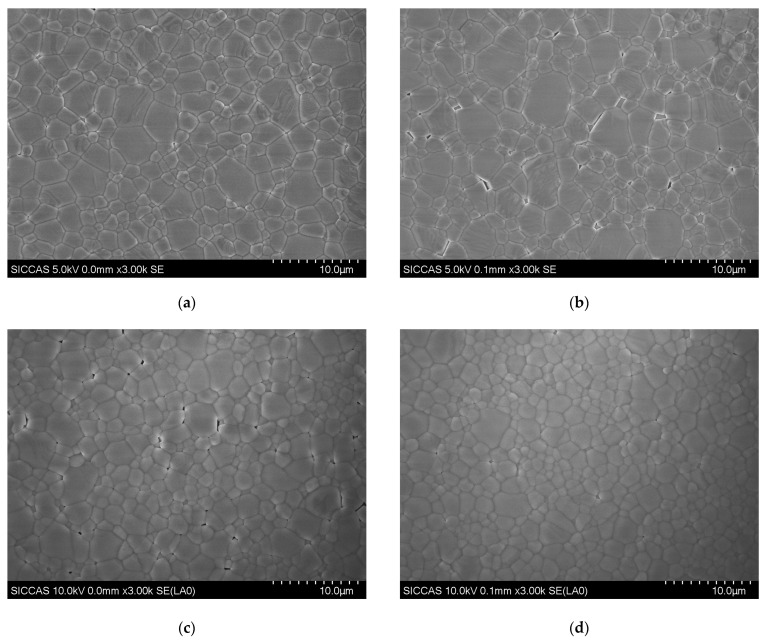

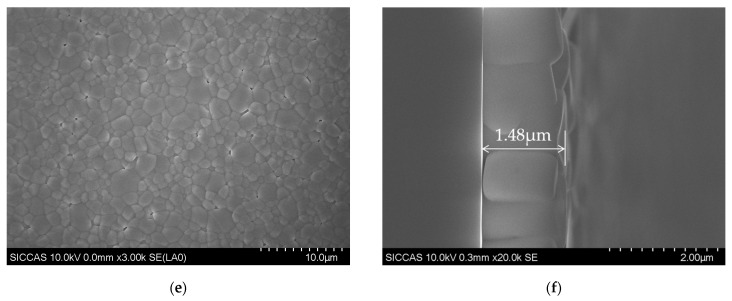
Surface (**a**–**e**) and cross-sectional (**f**) SEM images of KBr thin films grown at various substrate temperatures: (**a**) 50 °C, (**b**) 100 °C, (**c**) 150 °C, (**d**) 200 °C, and (**e**) 250 °C. (**f**) A representative cross-sectional SEM image of the film grown at 200 °C, showing its dense columnar structure.

**Figure 4:**
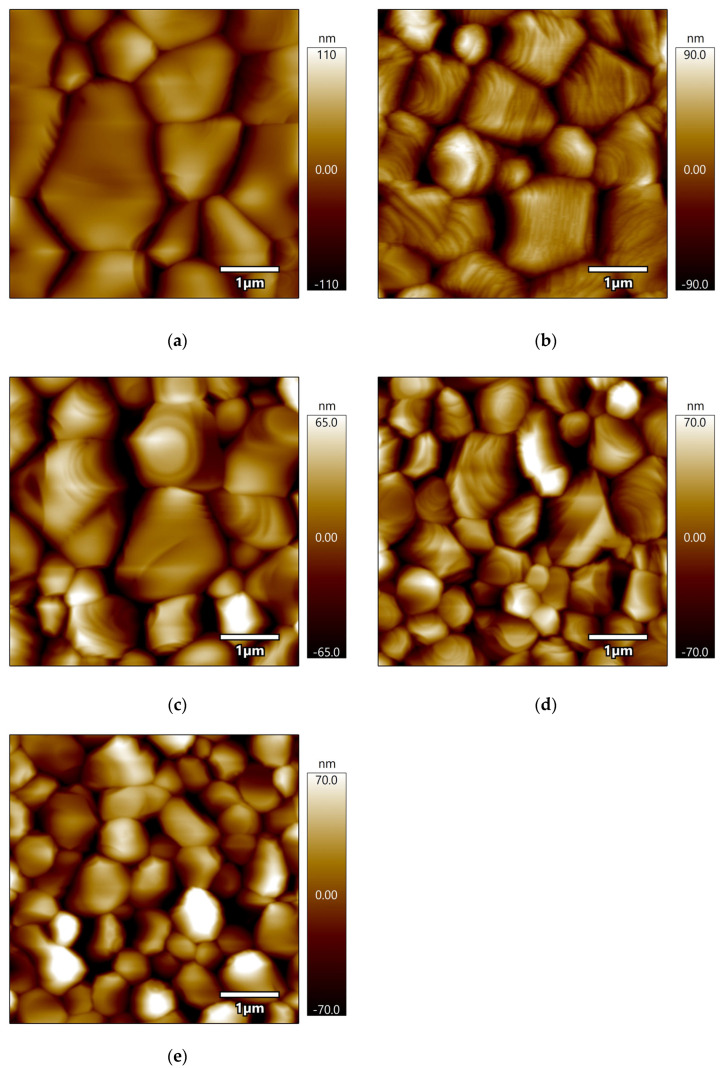
Surface topographies of KBr thin films grown at various substrate temperatures, imaged by AFM. (**a**) 50 °C, (**b**) 100 °C, (**c**) 150 °C, (**d**) 200 °C, and (**e**) 250 °C.

The surface morphology and topography of the KBr films, investigated by SEM (Figure 3) and AFM (Figure 4), undergo a complex evolution driven by substrate temperature. It is crucial to distinguish between the morphologically observed ‘grain size’ and the crystallographically determined ‘crystallite size’ (Section 3.1), as their comparison provides insight into the films’ internal structure. In the low-temperature regime (50–100 °C), film growth is dominated by nucleation and island coalescence. At 50 °C, limited adatom mobility results in a film composed of large, irregular, island-like domains with an internal average crystallite size of ~160 nm. Upon heating to 100 °C, a burst of new nucleation events causes these large islands to segment into finer grains, corresponding directly to the sharp decrease in the XRD-derived crystallite size to ~85 nm. However, this process leaves behind a network of wedge-shaped voids, slightly increasing the RMS roughness from 25.1 nm to 26.6 nm (Table 1).

The temperature range of 150–200 °C marks a critical transition toward film densification and planarization. At 150 °C, the film achieves its maximum surface smoothness (minimum RMS roughness of 24.6 nm) as enhanced surface diffusion planarizes the grain tops. Concurrently, SEM reveals that deep, narrow grooves at the grain boundaries remain, indicating that while the surface is highly planar, the film has not yet achieved full density. As the temperature rises to 200 °C, thermal energy promotes significant grain boundary migration and coalescence, healing the boundary voids and leading to a fully dense, uniform mosaic structure with a maximum crystallite size of ~113 nm. At the highest temperature of 250 °C, the film’s structural quality degrades. A combination of KBr re-evaporation and thermal stress triggers secondary nucleation and fragments larger grains, resulting in a re-refinement of the crystallite size to ~77 nm. This manifests as a honeycomb-like network of pits on the surface, causing the RMS roughness to increase sharply to 28.3 nm. This phenomenon, where high-temperature growth of simple columnar grains can be counteracted by complex degradation mechanisms, has been observed in analogous ionic crystal systems [27]. The cross-sectional SEM image of the dense film grown at 200 °C (Figure 3f) reveals a well-defined columnar structure, which is characteristic of films prepared by thermal evaporation. The measured thickness of approximately 1.48 µm is in excellent agreement with the nominal deposition thickness of 1.5 µm, confirming the accuracy of our process control.

In summary, the morphological analysis corroborates the findings from our structural study, identifying the 150–200 °C window as optimal for fabricating highly dense KBr films with smooth surfaces. The overall non-monotonic evolution of the film quality—improves from low to intermediate temperatures before degrading at higher temperatures due to the re-emergence of defects driven by re-evaporation and thermal mismatch [28]—provides a clear morphological basis for understanding and optimizing the films’ optical properties.

### 3.3. Optical Properties

**Figure 5:**
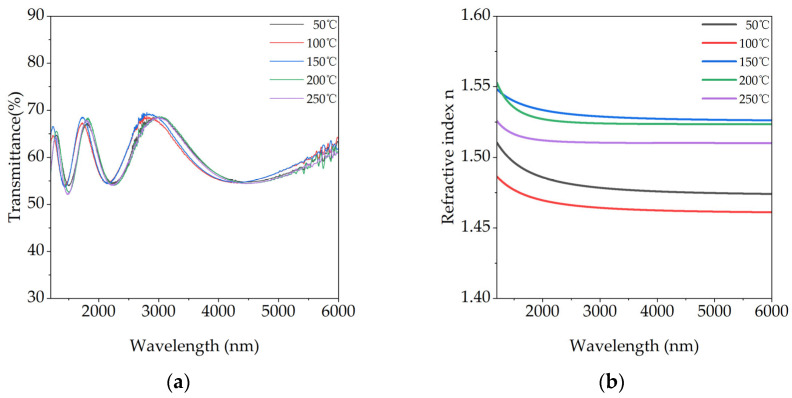
(**a**) Transmittance spectra of KBr films on Si substrates and (**b**) their corresponding refractive index (n) dispersion curves, for films grown at various temperatures.

**Figure 6:**
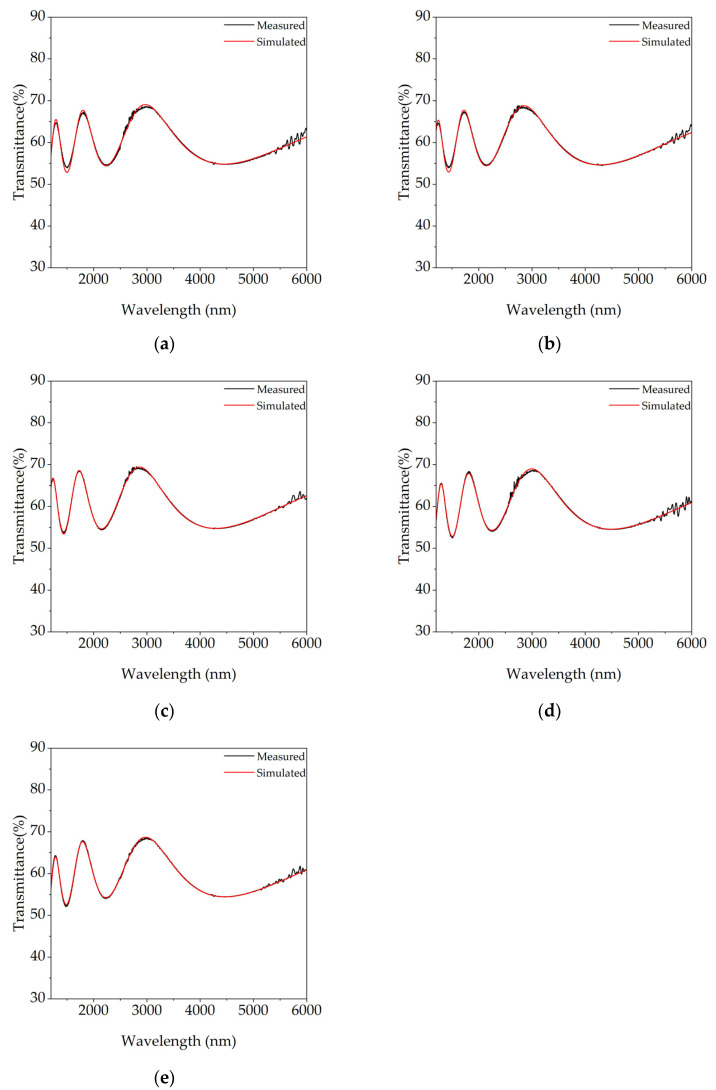
Comparison of the experimental transmittance spectra (measured, solid black lines) and the fitting theoretical spectra from the Cauchy model (simulated, solid red lines) for KBr films deposited at substrate temperatures of (**a**) 50 °C, (**b**) 100 °C, (**c**) 150 °C, (**d**) 200 °C, and (**e**) 250 °C.

Figure 5a presents the transmission spectra of KBr films deposited on silicon substrates over the 1.2–6 µm wavelength range. All curves exhibit clear, regular Fabry–Pérot interference fringes. The corresponding refractive index (n) dispersion curves, derived from the spectra via the Swanepoel envelope method, are shown in Figure 5b. As demonstrated in Figure 6, the theoretical spectra generated from the fitted model show excellent agreement with the experimental data for all samples, confirming the validity of the extracted refractive index. All films display normal dispersion characteristics. In agreement with the structural and morphological findings, films deposited at 150 °C and 200 °C exhibit the highest refractive indices, approaching the value of dense, bulk KBr [29]. This is a direct consequence of their high packing density, which is achieved through enhanced atomic diffusion and minimized porosity. In contrast, the film grown at 100 °C shows the lowest n value, attributed to a reduced effective packing density from wedge-shaped voids and micro-strain. The 50 °C and 250 °C samples exhibit intermediate refractive indices.

**Figure 7:**
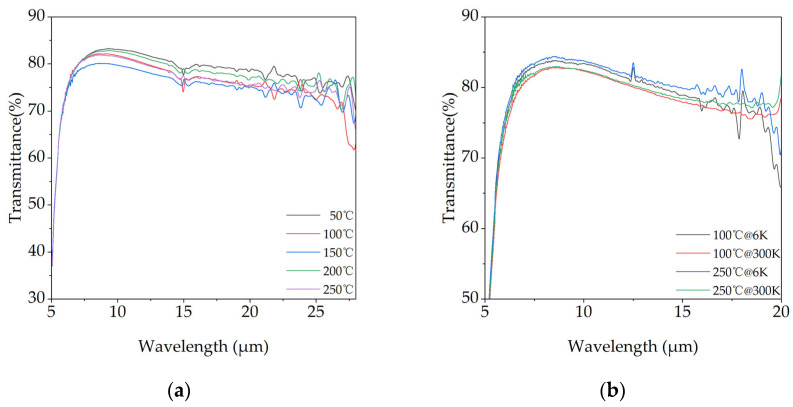
Infrared transmittance of KBr films on Diamond substrates. (**a**) Spectra of films prepared at different substrate temperatures in an ambient air atmosphere. (**b**) Comparison of the 100 °C and 250 °C samples measured at 300 K and 6 K under high vacuum.

Figure 7a illustrates the transmission spectra of films prepared on diamond substrates across the 5–28 µm spectral range. All samples demonstrate a broad transparent window in the mid- to long-wave infrared, with transmittance exceeding 75% between 6 and 25 µm, highlighting the potential of KBr as an infrared anti-reflection material. The transmittance varies non-monotonically with temperature, a behavior governed by a competition between scattering loss and Fresnel reflection loss. For the 50 °C film, the high overall transmittance is achieved because both primary loss mechanisms are suppressed: its lower effective refractive index, resulting from a high density of grain boundaries, reduces reflection loss, while a surface that is relatively free of fine-scale scattering centers minimizes scattering. As the temperature rises to 100 °C and 150 °C, the formation and expansion of voids and low-density regions at grain boundaries, as seen in SEM, intensifies volume (or bulk) scattering, which becomes the dominant loss factor and causes a decline in transmittance. At 200 °C, the film structure becomes maximally dense with significantly fewer defects, leading to minimal bulk scattering loss. Although densification increases the refractive index and thus reflection loss, the substantial reduction in scattering results in a sharp recovery of transmittance. However, at the excessively high temperature of 250 °C, the re-emergence of pores and grain boundary defects, coupled with increased surface roughness, reintroduces strong bulk and surface scattering, leading to a final deterioration in transmittance.

To further investigate the potential of these films for cryogenic applications, two representative samples (100 °C and 250 °C) were selected for transmittance measurements at both room temperature (300 K) and a deep cryogenic temperature (6 K). The results, displayed in Figure 7b, reveal a noticeable enhancement in transmittance for both films upon cooling. This improvement is attributed to the suppression of multi-phonon absorption. In ionic crystals like KBr, lattice vibrations (phonons) are the primary mechanism for infrared energy absorption, particularly in the long-wavelength region [30]. At room temperature, thermal energy excites a wide range of phonon modes. Cooling the film to 6 K drastically reduces this thermal energy, effectively “freezing” most phonon modes and thereby suppressing the multi-phonon absorption process, which directly increases the material’s transparency.

## 4. Conclusions

In this study, the influence of substrate temperature (50–250 °C) on the structural, morphological, and infrared optical properties of resistively evaporated KBr thin films was systematically investigated. The physical properties of the films were found to exhibit a complex and highly sensitive non-monotonic dependence on temperature. While increasing temperature promoted a strong <100> preferential orientation, key microstructural parameters—including crystallite size, dislocation density, and residual stress—underwent distinct non-monotonic evolutions. Morphological analysis further revealed that an optimal combination of high packing density (maximized at 200 °C) and surface smoothness (maximized at 150 °C) was achieved within the 150–200 °C range. These microstructural variations directly governed the film’s optical constants, leading to the highest refractive indices in this same temperature window. Furthermore, all films demonstrated significantly enhanced infrared transmittance at a cryogenic temperature of 6 K, confirming their potential for low-temperature optical systems. Based on a comprehensive evaluation of these interconnected properties, this work identifies the 150–200 °C range as the optimal processing window for fabricating high-quality KBr films. Films prepared in this window simultaneously possess a strong crystallographic texture, low defect density, and superior microstructural integrity, thereby achieving optical properties that closely approach theoretical bulk values.

By establishing quantitative relationships between a key deposition parameter and the multidimensional properties of KBr films, this research advances the fundamental understanding of alkali halide thin film growth. The findings also provide a critical experimental database and engineering guidance for the rational design and performance optimization of KBr-based optical components, including infrared windows, anti-reflection coatings, and filters. For practical applications, addressing the hygroscopic nature of these films is crucial. Future work could focus on protective strategies, such as encapsulating the KBr film with a thin, non-hygroscopic layer (e.g., BaF_2_), to enhance its durability while maintaining high infrared transmittance.

## Figures and Tables

**Table 1:** RMS roughness of KBr thin films deposited at different substrate temperatures.

Substrate Temperature (°C)	50	100	150	200	250
RMS Roughness (nm)	25.13	26.56	24.64	25.16	28.30

## Data Availability

The original contributions presented in this study are included in the article. Further inquiries can be directed to the corresponding author.
